# Supplementary figures and images for: Amyloid β induces hormetic-like effects through major stress pathways in a C. elegans model of Alzheimer’s Disease
Source: PLoS One. 2025 Apr 24;20(4):e0315810. doi: 10.1371/journal.pone.0315810 (PMC12021181; doi:10.1371/journal.pone.0315810)

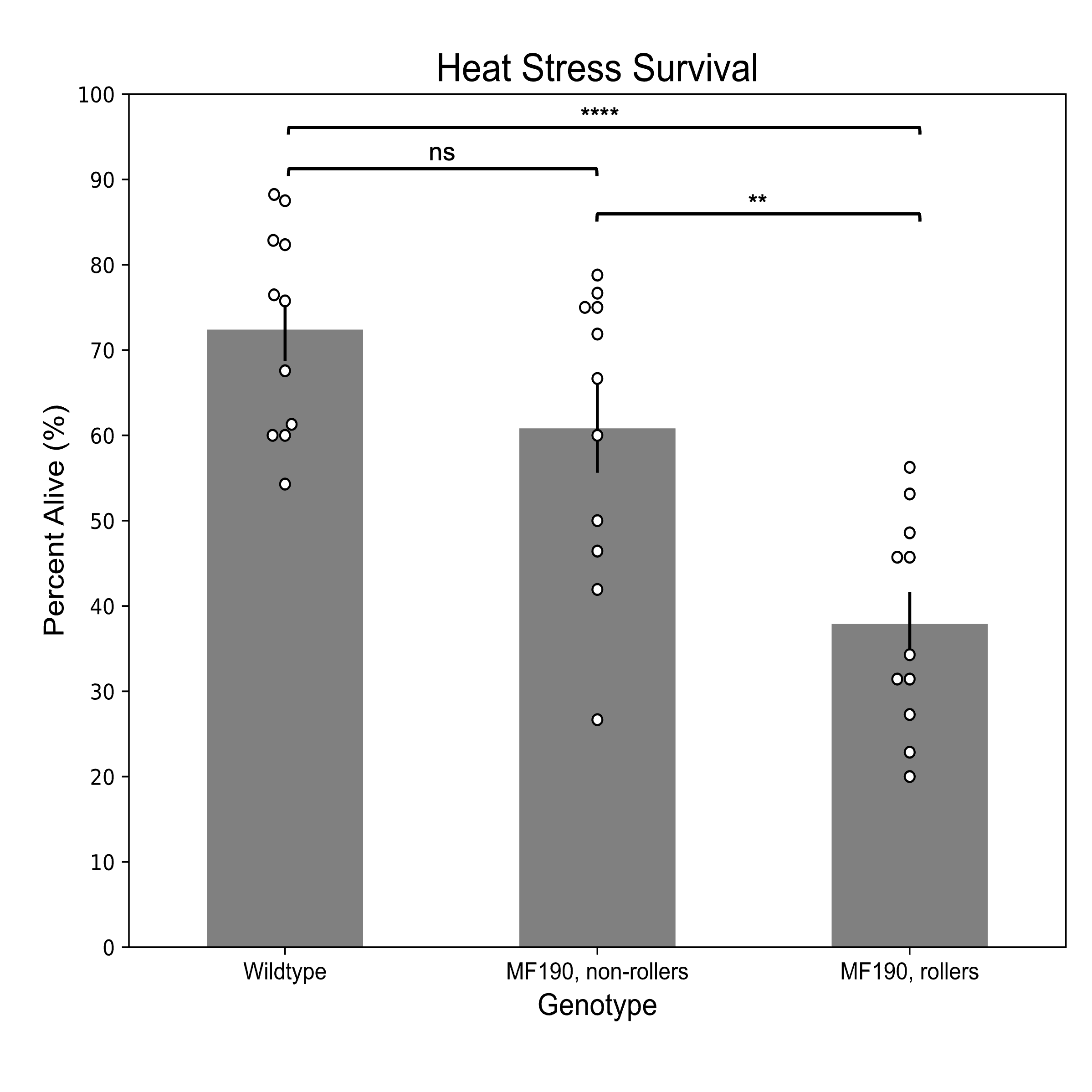

Supplement: S1 Fig — Survival rate after 4 hrs. at 37 °C heat stress exposure and overnight recovery at 20 °C. MF190 non-rollers do not exhibit a roller phenotype, while MF190 exhibit a roller phenotype. MF190 drives GFP in the PVD neuron by the des-2 promoter, and also contains a rol-6(su-1006) rescue. (TIF) [file pone.0315810.s001.tif]
